# Endosalpingiosis of the Gallbladder: A Unique Complication of Ruptured Ectopic Pregnancy

**DOI:** 10.7759/cureus.5393

**Published:** 2019-08-15

**Authors:** Katherine R Porter, Charanjeet Singh, Vladimir Neychev

**Affiliations:** 1 Miscellaneous, University of Central Florida College of Medicine, Orlando, USA; 2 Pathology, AdventHealth Winter Park, Winter Park, USA; 3 Surgery, University of Central Florida College of Medicine, Orlando, USA

**Keywords:** cholecystitis, tubal pregnancy, invasion-migration

## Abstract

A 60-year-old woman with a history of a ruptured ectopic pregnancy and subsequent salpingo-oophorectomy presented with clinical signs. Pre-operative imaging and intra-operative observations were highly suggestive of acute on chronic cholecystitis. A laparoscopic cholecystectomy was performed. In addition to confirming calculous cholecystitis, final pathology revealed endosalpingiosis on the serosal surface of the gallbladder. Endosalpingiosis is a rare, benign presence of glands lined by tubal-like epithelium, and the few case reports describe it on the surface of the female reproductive organs or seeded on the pelvic peritoneum. We hypothesize that, in this unique case, the endosalpingiosis is due to patient’s ruptured ectopic pregnancy, which allowed tubal epithelial cells to spread to the gallbladder. The only documented cases of endosalpingiosis outside the pelvic and lower abdominal organs have been congenital choristomas. To our knowledge, this is the first documented case of acquired endosalpingiosis of the gallbladder.

## Introduction

Endosalpingiosis is the benign presence of glands lined by tubal-like epithelium, which contains ciliated columnar cells, nonciliated columnar secretory mucus cells and intercalary peg cells in tissues outside the fallopian tubes. The condition usually manifests in organs contained by the pelvic and lower abdominal peritoneum and is often asymptomatic [1]. There are several theories as to the pathogenesis of endosalpingiosis, including both embryologic and acquired routes. The only cases of endosalpingiosis reported outside of the pelvis and lower abdomen are developmental choristomas in the mediastinum, spleen, and umbilicus [2, 3, 4-7]. Here, we present a unique case of endosalpingiosis of the gallbladder, most likely acquired secondary to a ruptured ectopic pregnancy, discovered incidentally during an elective cholecystectomy.

## Case presentation

A 60-year-old caucasian woman with a past medical history significant for obesity, hypertriglyceridemia, hypertension, gallstones, and gastroesophageal reflux disease was evaluated for worsening postprandial right upper abdominal discomfort, bloating, nausea, and mild to moderate pain radiating to the back. Her surgical history was significant for an emergency right salpingo-oophorectomy for a ruptured ectopic pregnancy roughly 30 years. The vital signs and physical exam were unremarkable.

**Figure 1:**
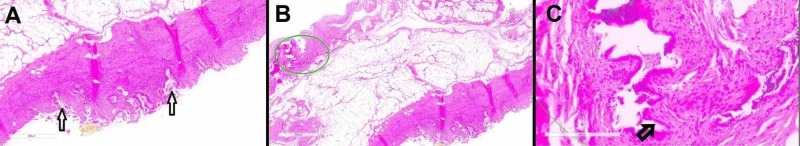
Histological studies of the gallbladder consistent with endosalpingiosis A - Mucosal surface of gall bladder (40x), showing mild chronic cholecystitis. Arrows are showing the mucosal epithelium extending into the hypertrophied smooth muscle layer. B - Circled is an area on the non-peritonealized surface, opposite to gall bladder mucosa (20x). The circled area shows two acinar structures lined by simple cuboidal epithelium. C - Arrow shows cuboidal epithelium of acinar structure (200x) with a brush border and terminal bar, which are consistent with endosalpingiosis in this female patient. No Mullerian type stroma was seen.

Abdominal ultrasonography showed gallstones without cholecystitis, confirmed by a hepatobiliary iminodiacetic acid (HIDA) scan. The preoperative diagnosis was acute on chronic calculous cholecystitis. A detailed discussion of the natural history, possible causes, and management of gallstone disease, including operative versus nonoperative management, was carried out, and the decision was made to proceed with surgery. Informed consent was obtained, and a laparoscopic cholecystectomy was performed without complications. During surgery, multiple adhesions of the transverse colon and omentum to the anterior and right lateral abdominal wall were noted, and lysis of adhesions was performed. The post-operative diagnosis remained acute on chronic calculous cholecystitis (Figure 1 A). Surgical pathology confirmed cholecystitis with a surprising finding of endosalpingiosis focus on the serosal surface of the gallbladder (Figures 1 B and C).

The patient tolerated and recovered from surgery well and was discharged to home on the same day of surgery.

## Discussion

The incidence and prevalence of endosalpingiosis are poorly studied. This could be in part due to its asymptomatic nature or coexistence with endometriosis. It is possible that tubal epithelial cells are lost during the surgical ablation of endometriosis prior to histological examination [1]. In a study of 32 breast cancer (BRCA) positive patients who underwent risk-reducing salpingo-oophorectomies, seven of 32 patients had cytologic evidence of endosalpingiosis in the peritoneal washings taken intraoperatively, indicating the possibility of tubal epithelial shedding prior to and during these types of surgeries [8].

There are several proposed theories of the pathogenesis of endosalpingiosis, and these theories fall into two categories: embryological origin and acquired origin. It was proposed that endosalpingiosis and the other three Mullerian diseases - endometriosis, endocervicosis, and adenomyosis - arise as choristomas, normal Mullerian tissues, singly or in combination, incorporated within other normal organs during organogenesis. Embryonic endosalpingiosis most often results in cystic, often painful lesions in the affected areas [9].

The ability of tubal endometrial cells to proliferate outside the fallopian tube was first postulated by Sampson in 1930, who observed the invasion of fallopian tube epithelium through the walls of post-salpingectomy tubal stumps [10]. Kistner described five theories for the development of endosalpingiosis after menarche: (1) intra-operative transplantation of the tubal mucosa to peritoneal surfaces, (2) direct expansion of the tubal epithelium and adhesion to the peritoneum, (3) differentiation of multipotential coelomic stem cells into oviduct epithelium and resultant metaplasia, (4) excessive tubal proliferation secondary to salpingitis or other cell injury, and (5) metastatic spread via the lymphatic or vascular systems. Contrary to embryonic endosalpingiosis, acquired endosalpingiosis is most often, not discernable macroscopically [1].

We believe the gallbladder endosalpingiosis presented in this case is likely due to the patient’s ruptured ectopic pregnancy 30 years ago, and her subsequent salpingo-oophorectomy. Although embryonic relationship between tubal epithelium and the gallbladder serosa in this case is not completely impossible, it is highly unlikely. The endosalpingiosis did not produce symptoms, and its presence was not evident on intra-operative or post-operative gross examination of the gallbladder. These indications, and the history of ruptured ectopic pregnancy, all point to an acquired route of pathogenesis.

It is our hypothesis that our patient’s ruptured ectopic pregnancy resulted in hemorrhagic dissemination of the tubal epithelial cells through the abdominal cavity, where they seeded the gallbladder. This hypothesis is also supported by the evidence of extensive adhesions found localized in the right flank and right upper abdominal quadrant during surgery. The vast distance the cells traveled to seed the gallbladder is the main limitation of this hypothesis. To our knowledge, this is the first case of endosalpingiosis of the gallbladder documented in the literature.

Given this hypothesis, it is plausible that there are other areas of endosalpingiosis present within the patient’s abdominal cavity. However, due to the asymptomatic nature of these lesions, it would go against the laws of beneficence and non-maleficence to search for them. Although this diagnosis bore a little impact on the patient, this incidental finding demonstrated the resilience and the potential of differentiated human cells to thrive outside their native tissue niche. This unique case may stimulate the establishment of novel cellular models guiding the development of new testable hypotheses for better understanding of the global principles and programs of the epithelial-to-mesenchymal transition and associated cellular invasion-migration programs.

## Conclusions

As the first documented case of endosalpingiosis of the gallbladder, this case increases awareness of human cells’ propensity to survive and thrive, even in the most foreign of conditions. Endosalpingiosis remains difficult to study because of its traditionally asymptomatic nature. However, since the different forms of mullerianosis, including endometriosis and endocervicosis, often occur together, it is imperative that clinicians use every case of as an opportunity to develop a greater understanding about this group of diseases as a whole.
